# Correlation between clinicians-assigned weights to findings and their diagnostic odd ratio; case of congestive heart failure

**DOI:** 10.1186/s40200-016-0262-6

**Published:** 2016-09-23

**Authors:** Akbar Soltani, Farzane Saeidifard, Abbasali Keshtkar, Fatemeh Shakki Katouli

**Affiliations:** 1 Evidence-Based Medicine & Critical Thinking Working Team, Endocrinology and Metabolism Research Institute, Tehran University of Medical Sciences, Tehran, Iran; 2 Endocrinology and Metabolism Research Center, Endocrinology and Metabolism Clinical Sciences Institute, Tehran University of Medical Sciences, Tehran, Iran; 3 Endocrinology and Metabolism Clinical Sciences Institute of Tehran University of medical sciences, EMRI, Tehran, Iran; 4 Radiology Department of Shariati Hospital, Tehran University of Medical Sciences, Tehran, Iran

**Keywords:** Congestive heart failure, Evidence based medicine, Likelihood ratio

## Abstract

**Background:**

Incorrect estimation of pretest probability and misinterpretation of test results can change post-test probability in medical decision making. The aim of this study was to evaluate how physicians assess weight of findings of congestive heart failure (CHF) and how much their estimation is correlated with findings’ Diagnostic Odd Ratio (DOR).

**Methods:**

The participants were asked to answer a questionnaire based on a scenario of a patient having dyspnea. Eighteen findings in 3 categories including: history, examination and radiographic findings were inserted along a column and a row as a matrix. The respondents had to compare each finding in the column with all other findings in the row and insert a mark in boxes below the findings of the row that had greater weight compared to the finding in the column. The weight of each finding was considered as total number of “marked boxes” in front of that finding. DOR of findings was calculated using their positive and negative likelihood ratios (LRs) based on current best evidence. Findings ranked in the order of their DOR and were compared with the ranking in the order of participants-assigned weights. We examined correlation between average weights assigned by physicians and DOR of findings. In subgroup analysis correlations between average weights assigned by physicians and DOR of history, examination and radiographic findings were examined.

**Results:**

Seventy five physicians completed the questionnaire. Correlation between ranking in the order of findings’ DOR and ranking in the order of clinicians-assigned weights was significant (*p*-value = 0.005 *r* = 0.64). In contrast correlations between participants-assigned weights and DOR of history, examination and radiographic findings were positive but non- significant (*r* = 0.181, *p*-value = 0.7, *r* = 0.343, *p*-value = 0.506 and *r* = 0.219, *p*-value = 0.723 respectively).

**Conclusion:**

Our result show that although correlation between clinicians-assigned weights and DOR of entire findings was significant, correlations between clinicians-assigned weights to the different categories of findings and their DOR were not significant. Reevaluating probabilistic reasoning by emphasis on using LRs can make pretest probability estimating and interpretation of test results more objective and would ultimate in more precise and homogenous post-test probabilities.

## Background

Diagnostic errors are multi-factorial and could be categorized into 3 types: 1. Patient-related errors: due to atypical manifestation of disease. 2. Health service-related errors: due to defects in health services 3. Cognitive Errors or physician-related errors: due to mistakes in data collecting, lack of knowledge or impaired reasoning [1–3]. In order to decrease cognitive errors, probabilistic diagnostic reasoning based on Bayes theorem, which is also called as threshold approach, was integrated into evidence-based medicine (EBM) to provide the best and the most relevant approach for clinical practice [4]. According to the threshold approach there are 3 steps in diagnostic process: pretest probability estimating, using likelihood ratio (LR) of tests and post test probability estimating. Primary estimation of a disease’s probability that is called pretest probability is combined with LR of diagnostic tests. The outcome is post-test probability [5].^1^ Error in pretest probability estimating or using LR of tests leads to error in estimation of post-test probability and subsequent decision(s). Overestimation of the pretest probability results in unnecessary or invasive treatments and underestimating the prior probability leads to misuse of diagnostic tests or not treating the patient [6, 7]. Estimation of pretest probability depends on clinician’s intuition and judgment about the disease and disease’s prevalence [8–13]. The first component makes estimation of pretest probability subjective that leads to heterogeneity and controversy over the resulted post-test probability [6, 13].

Besides the studies that have indicated on the importance of the second step (using LR of tests) in probabilistic reasoning, several studies have indicated on the importance of the first step or pretest probability estimation by the physicians, residents and medical students [9, 12, 14, 15] and their difficulties in estimating both pretest probability and likelihood ratio of tests [6, 7, 16–18]. In these studies clinicians had to approach to a scenario and estimate the likelihood of some differential diagnoses. This approach could detect the discrepancies in estimation of pretest probability of disease but not address the details and underlying causes.

Owing to high prevalence and burden of congestive heart failure (CHF), a patient with CHF was chosen. The aim of this study was to evaluate how experts and newly graduated physicians estimate weight of clinical and radiographic findings in approaching a patient suspected of CHF and how much their estimation correlated with findings’ actual weight based on their LR.

## Method

### Study population and design

This cross-sectional study was done during the international CHF symposium, which was held for general practitioners, internists, cardiologists and pulmonologists in Tehran by Shahid Beheshti University of Medical Sciences. Participants were asked to answer a pre-prepared questionnaire. They had to complete the questionnaire based on their estimation of weight of findings in diagnosis of CHF.

### Clinicians-assigned weights to each clinical finding

The questionnaire had two parts. The first part included questions regarding the participants’ specialty, academic affiliation and number of years working as a clinician. The second part consisted of a scenario of a patient having dyspnea who was admitted to emergency department to be examined for CHF and a matrix with 18 findings in 3 categories including: 1.History findings: history of heart failure, myocardial infarction, coronary artery disease, paroxysmal nocturnal dyspnea, orthopnea, edema, dyspnea on exertion. 2. Examination findings: third heart sound, jugular venous distension, abdominojugular reflux, rales, any murmur, lower-extremity edema. 3. Radiographic findings: pulmonary venous congestion, interstitial edema, alveolar edema, cardiomegaly, and pleural effusions in addressing the CHF.

The matrix was a table that was used to determine the relative priority of findings. In this matrix all the findings were listed in the first column as reference findings. The same findings were repeated in a row as comparative finding. A box to make a mark was assigned below each finding of the row in front of the reference findings. The participants had to make a mark below those findings that had lesser weight in comparison with the reference finding so the reference finding had greater weight in comparison with the marked findings in the row and lesser weight in comparison with findings with empty boxes. The weight of each reference finding is calculated by total number of “marked boxes” in front of that finding.

We checked accuracy and precision of all the questionnaires in terms of weighing of the findings. As an example if in a completed questionnaire the finding A had greater weight in comparison with the finding B and the finding B had greater weight compared to the finding C, so the finding A had to have greater weight in comparison with the finding C.

An example of this matrix is shown below. In this matrix the participant has compared the history of heart failure, as reference finding, with all other findings as comparative findings in diagnosis of heart failure and has made a mark in boxes below the “third heart sound, rales, interstitial edema and pleural effusions”. It means that History of Heart failure has more weight in comparison with mentioned findings in diagnosis of CHF. The weight of “history of heart failure” in diagnosis of CHF would be 4.

**Table:** 

Reference finding	Comparative findings																	
	History of Heart failure	Myocardial infarction	Coronary artery disease	Paroxysmalnocturnal dyspnea	Orthopnea	Edema	Dyspnea on exertion	Third heart sound	Abdominojugular reflux	Jugular venous distension	Rales	Any murmur	Lower-extremity edema	Pulmonary venous congestion	Interstitial edema	Alveolar edema	Cardiomegaly	Pleural effusions
History of Heart failure								×			×				×			×

### Sensitivity, specificity and likelihood ratio of each clinical finding

We obtained positive and negative LRs (LR+ and LR- respectively), Sensitivity and Specificity of each finding from current best evidence. Database of Medline was searched through Pubmed search engine using following key words :“left sided heart failure”[Mesh] OR “congestive heart failure”[Mesh] combined with “diagnostic accuracy” OR “physical examination” OR “medical history taking” OR “sensitivity and specificity” OR “Bayes Theorem” to identify potentially-relevant articles. A systematic review published by JAMA in 2005 has reported the Sensitivity, Specificity and LRs of each finding [19, 20]. These reported characteristics were used as reference and clinicians- estimated weights were compared with them.

### Statistical analysis

The data were analyzed using SPSS version 16. We calculated an average weight for each finding based on participants-assigned weights and ranked them in the order of their average weight. Diagnostic Odds Ratio (DOR) was calculated for each finding based on its LRs. DOR index generally indicates the efficacy or potency of a finding [21]. Findings ranked in the order of DOR and were compared with the ranking in the order of participants-assigned weights.

$$ \mathrm{D}\mathrm{O}\mathrm{R}\kern0.5em =\kern0.5em \mathrm{T}\mathrm{P}*\mathrm{T}\mathrm{N}/\mathrm{F}\mathrm{P}*\mathrm{F}\mathrm{N} $$

To calculate DOR based on LR+, LR-, Sensitivity and Specificity, following formulas are available [21]:

$$ \mathrm{D}\mathrm{O}\mathrm{R}\kern0.5em =\kern0.5em \mathrm{S}\mathrm{e}\mathrm{n}*\mathrm{S}\mathrm{p}\mathrm{e}/\left(1\hbox{-} \mathrm{S}\mathrm{e}\mathrm{n}\right)*\left(1\hbox{-} \mathrm{S}\mathrm{p}\mathrm{e}\right) $$

$$ \mathrm{D}\mathrm{O}\mathrm{R}\kern0.5em =\kern0.5em \mathrm{P}\mathrm{P}\mathrm{V}*\mathrm{N}\mathrm{P}\mathrm{V}/\left(1\hbox{-} \mathrm{P}\mathrm{P}\mathrm{V}\right)*\left(1\hbox{-} \mathrm{N}\mathrm{P}\mathrm{V}\right) $$

$$ \mathrm{D}\mathrm{O}\mathrm{R}\kern0.5em =\kern0.5em \mathrm{L}\mathrm{R}+/\mathrm{L}\mathrm{R}\hbox{-} $$

2

We analyzed correlation between average weights assigned by physicians and DOR of findings. In subgroup analysis correlations between average weights assigned by physicians and DOR of history finding, examination finding and radiographic findings were examined separately. Correlations between the average weights assigned by faculty members and non-faculty members, specialists and subspecialists and expert and novice physicians were analyzed separately using independent sample t-tests. (Expert: was defined as a physician who had clinical experience more than 6 years. Novice: Was defined as a physician who had clinical experience of 6 years or less.)

## Result

Seventy five completed questionnaires out of 200 questionnaires were returned (37.5 %). Thirty six (48 %) out of 75 were expert and 39 (52 %) were novice, 68 (90 %) were specialist and seven were subspecialists, and also 27 (36 %) were faculty member while 48 (64 %) were non-faculty member.

Alveolar edema, cardiomegaly and interstitial edema achieved the three highest average weights by participants (13.6 ± 3.4, 12.9 ± 3.9, 12.1 ± 3.0 respectively) whereas pulmonary venous congestion, interstitial edema and dyspnea on exertion had highest DOR (Table 1).

**Table 1:** Findings of CHF and their characteristics, ranking in the order of findings’ calculated DOR and in the order of the average of clinicians-assigned weights

Findings	Sensitivity	Specificity	LR+	LR-	DOR	Ranking1^a^	Ranking2^b^	Mean+/-SD
History findings								
Heart failure	0.60	0.90	5.8	0.45	12.9	3	13	6.7+/-4.8
Myocardial infarction	0.40	0.87	3.1	0.69	4.5	10	17	4.4+/-3.2
Coronary artery disease	0.52	0.70	1.8	0.68	2.65	18	18	3.5+/-3.5
Paroxysmal nocturnal dyspnea	0.41	0.84	2.6	0.70	3.7	12	11	8.3+/-3.5
Orthopnea	0.50	0.77	2.2	0.65	3.4	14	12	8.1+/-3.0
Edema	0.51	0.76	2.1	0.64	3.3	15	16	4.9+/-2.4
Dyspnea on exertion	0.84	0.34	1.3	0.48	2.71	17	15	6.2+/-2.2
Examination findings								
Third heart sound	0.13	0.99	11	0.88	12.5	4	8	8.9+/-3.9
Abdominojugular reflux	0.24	0.96	6.4	0.79	8.1	6	9	8.9+/-2.6
Jugular venous distension	0.39	0.92	5.1	0.66	7.7	7	10	8.6+/-2.5
Rales	0.60	0.78	2.8	0.51	5.5	9	6	9.2+/-3.6
Any murmur	0.27	0.90	2.6	0.81	3.2	16	14	6.2+/-4.7
Lower-extremity edema	0.50	0.78	2.3	0.64	3.6	13	5	9.5+/-3.4
Radiographic findings								
Pulmonary venous congestion	0.54	0.96	12.0	0.48	25.0	1	4	13.3+/-12.1
Interstitial edema	0.34	0.97	12.0	0.68	17.7	2	3	12.1+/-3.0
Alveolar edema	0.06	0.99	6.0	0.95	6.3	8	1	13.6+/-3.4
Cardiomegaly	0.74	0.78	3.3	0.33	10.0	5	2	12.9+/-3.9
Pleural effusions	0.26	0.92	3.2	0.81	4.0	11	7	9.1+/-5.5

^a^-Ranking in the order of DOR

^b^-Ranking in the order of the average of clinicians-assigned weights

History of coronary artery disease and history of myocardial infarction ranked as the 2 least important findings by participants, whereas according to the ranking in the order of findings’ DOR, 2 findings with the least importance were the History of coronary artery disease and Dyspnea on exertion (Table 1).

We analyzed correlation between the ranking of findings in the order of their DOR and the ranking in the order of participants-assigned weights. This correlation was positive and statistically significant (*ρ* = 0.64, *p*-value = 0.005). In subgroup analysis correlation between average of participants-assigned weights and DOR of history findings, examination findings *p*-value = 0.506 and *ρ* = 0.219, *p*-value = 0.723 respectively). Correlations between weights assigned by different groups of physicians (experts, novices, faculty members and non-faculty members) and DOR of different categories of findings are shown in Table 2.

**Table 2:** Correlation between weights assigned by different groups of physicians to the findings of CHF and DOR of different categories of findings

Categories of findings	Groups of physicians	Correlation coefficient (spearman’s rho)	*p*-value
History findings	Experts	0.286	0.534
	Novices	0.075	0.873
	Faculty members	0.419	0.439
	Non-faculty members	-0.102	0.827
Examination findings	Experts	0.527	0.283
	Novices	0.179	0.734
	Faculty members	0.758	0.081
	Non-faculty members	-0.251	0.631
Radiographic findings	Experts	0.653	0.232
	Novices	0.180	0.772
	Faculty members	0.236	0.702
	Non-faculty members	0.616	0.286

Correlations between the weights assigned by experts and novices and also between faculty members and non-faculty members to different categories of findings are shown in Table 3.

**Table 3:** Correlation between the weights assigned by different groups of physicians to the different categories of findings of CHF

	Expert with novice (correlation coefficient, *p*-value)	Faculty member with non-faculty member (correlation coefficient, *p*-value)
History findings	*ρ* ^*^ = 0.937, *P*-value =0.002	*ρ* ^*^ = 0.827, *P*-value = 0.023
Examination findings	*ρ* ^*^ = 0.830, *P*-value = 0.041	*ρ* ^*^ = 0.296, *P*-value = 0.569
Radiographic findings	*ρ* ^*^ = 0.727, *P*-value = 0.164	*ρ* ^*^ = 0.589, *P*-value = 0.296

^*^Spearman’s rho

Significant correlations have been demonstrated between weights assigned by experts and novices to the history and examination findings and between faculty members and non-faculty members to the history findings.

## Discussion

While managing a patient, physicians seek future findings in accordance with and following to previous findings. When all the findings are presented simultaneously, clinicians compare each finding with all the findings of all groups (history, examination and radiographic findings). Giving primacy to the findings in this situation would be easier than the situation that clinicians face a particular group of findings and make comparison between the findings of that specific group. As in our subgroup analysis correlations between clinicians-assigned weights and DOR of different categories of findings were not statistically significant. These results suggest that clinicians’ estimation of weight of findings of each group is not correlated with their actual value based on findings’ LRs. This lack of correlation could be interpreted as mismanagement of different steps of diagnostic process based on Bayes theorem including pretest probability estimation and test interpretation that could be ended in wrong estimation of post-test probability. These result are in accordance with previous studies in this area [6, 10, 12, 16–18, 22].

Estimation of weight of radiographic findings in this study was as the second step of the threshold approach in diagnostic process. In regard to the importance of this part of diagnosis [23–25] incorrect estimation of weight of radiographic findings and lack of correlation between clinicians-estimated weights and DOR of these findings distort the estimation of post-test probability. But this step is affected by the estimation of primary probability that is somewhat subjective and more complicated, because according to Bayes theorem, estimation of primary probability depends on prevalence of diseases in clinical setting and also clinicians’ intuition about patient. While it has been shown that estimation of primary probability by physicians is usually not exact and there is a great deal of variation among them in terms of estimation of primary probability. This variation has been demonstrated among different expertise levels and different medical conditions [9, 12, 14, 15, 26]. These studies have been focused on the estimation of primary probability of a particular disease as main issue while the present study has focused on underlying cause of incorrect estimation of pretest probability and it has been clarified that physicians are not able to weigh clinical findings accurately according to their category and their position in the diagnostic process.

Surprisingly, there was a significant correlation between the weights assigned by experts and novices to the history and examination findings whereas correlation between the weights assigned by these two groups of clinicians to the radiographic findings was not statistically significant. However weights assigned by these groups of clinicians were not correlated with the DOR of different groups of findings (Table 3). Although Allen et al. have reported that experts in comparison with novices are more successful in producing hypothesis, choosing appropriate reference and solving inconsistencies as result of their past knowledge and experience [13], these items indicate their superiority in making differential diagnoses. If these qualifications were not combined with updated evidence may cause errors in estimating actual weight of findings. As a result the same mistake is made in ruling in or out of a diagnosis among expert and novice clinicians that is equal to making mistakes in estimation of pretest probability as well as one study showed that experience did not decrease the variance of primary probability estimation [15].

Another result of this study was the differences between faculty members and non-faculty members. Although there was not any significant correlation between the weights assigned by faculty members and non-faculty members and DOR of different groups of findings, correlations between the weights assigned by faculty members and DOR of history and examination findings were stronger than non-faculty members (r(FM) = 0.419 vs. r(NFM) = - 0.102 and r(FM) = 0.758 vs. r(NFM) = -0.251 respectively).^3^ On the other hand correlation between weights assigned by non-faculty members and DOR of the radiographic findings was stronger than correlation between weights assigned by faculty members and DOR of radiographic finding (r(NFM) = 0.616 vs. r(FM) = 0.236). Although the sample size was low, it can be hypothesized that due to educational curriculum in teaching hospitals faculty members mostly emphasize on the value of findings of history taking and physical examination in their medical students training. Non-faculty members do not have this position and since most of their work time is spent in their private office, they are mostly dependent on laboratory and radiographic findings and may ignore the value of history and examination finding in their medical practice.

According to our study there was no difference between experts and novices in assigning weight to clinical findings but it cannot be a satisfactory reason to ignore other differences between experts and novices e.g. experts try to use several diagnostic approaches like pattern recognition in their diagnosis [27, 28]. Response rate to our questionnaire was 37.5 % that decreases the power of the study. Participants’ unfamiliarity with different design of this questionnaire might be a cause. Negative or non-significant correlations demonstrated between different groups of clinicians and different categories of findings would be due to low sample size of each group of clinicians. It is not obvious whether it is possible to generalize the result of this study to other clinical conditions of other specialties.

We concluded that although correlation between clinicians-assigned weights and DOR of entire findings was significantly positive, correlation between the weights assigned by clinicians to different categories of findings of CHF and their DOR was not statistically significant. Reevaluating probabilistic reasoning by emphasis on using LRs of clinical and para-clinical findings can make pretest probability estimation and interpretation of test results more objective and ultimate in more precise and homogenous post-test probability.

## Conclusion

As a conclusion, there is a significant correlation between weights assigned by experts and novices to the history and examination findings and between faculty members and non-faculty members to the history findings.

Also, a significant positive correlation between the weights that clinicians assigned to the findings of CHF and DOR of findings. This means that clinicians were able to rank the findings were presented simultaneously, in an acceptable order whereas the situation of practice is completely different.

Our survey encountered some limitations that deserve comments. The findings were not categorized so the clinicians did not have opportunity to use their EBM skills. Using clinical scenarios, like which had been used in our questionnaire, might make experts use their prior knowledge based on text books instead of using best current evidence or their clinical experience.

## Abbreviations

CHF: Congestive heart failure
DOR: Diagnostic Odd Ratio
EBM: Evidence-based medicine
FM: Faculty member
FN: False negative
FP: False positive
LR: Likelihood Ratio
NFM: Non-faculty member
NPV: Negative predictive value
PPV: Positive predictive value
Sen: Sensitivity
Spe: Specificity
TN: True negative
TP: True positive.

## Footnotes

1
$$ \mathrm{pretest}\kern0.5em \mathrm{odds}\kern0.5em =\kern0.5em \frac{pretest\kern0.5em  probability}{1- pretest\kern0.5em  probability} $$

$$ \mathrm{posttest}\kern0.5em \mathrm{odds}\kern0.5em =\kern0.5em  pretest\kern0.5em  odds\kern0.5em \times \kern0.5em  likelihood\kern0.5em  ratio\kern0.5em  of\kern0.5em  tests $$

$$ \mathrm{posttest}\kern0.5em \mathrm{probability}\kern0.5em =\kern0.5em \frac{posttest\kern0.5em  odds}{posttest\kern0.5em  odds\kern0.5em +\kern0.5em 1} $$

2 TP: True positive, TN: true negative, FP: false positive, FN: false negative, PPV: positive predictive value, NPV: negative predictive value, Sen: sensitivity, Spe: specificity

3 FM: faculty member NFM: non-faculty member
